# Supplementary material for: Excess Zinc Alters Cell Wall Class III Peroxidase Activity and Flavonoid Content in the Maize Scutellum
Source: Plants (Basel). 2021 Jan 21;10(2):197. doi: 10.3390/plants10020197 (PMC7909774; doi:10.3390/plants10020197)
Supplement: Supplementary file 1 [file plants-10-00197-s001.pdf]

plants

MDPI

Original Research

Zinc Induces Cell Wall Stress and Changes in Class III Peroxidase Activity and Flavonoid Concentration

Díaz-Pontones David Manuel., Corona-Carrillo José Isaac., Herrera-Miranda Carlos., González Sandra.

Department of Health Sciences, Division of Biological and Health Sciences,
Laboratory for Tissue Biochemistry, Universidad Autónoma Metropolitana-Iztapalapa, México City, México
Avenida San Rafael Atlixco No 186 Col Vicentina Iztapalapa CP 09340 Ciudad de México
dmdp@xanum.uam.mx

Supplements

TABLE S1. GROWTH REGRESSION MODEL				
CONDITION	ROOT		PLUMULE	
	ECUATION	R^2	ECUATION	R^2
H_2O	y = 4.8055ln(x) - 14.642	R^2 = 0.9777	$y = 0.0023x^{1.748}$	R^2 = 1
H_2O+H^+	y = 4.9644ln(x) - 15.042	R^2 = 0.9969	$y = 0.0024x^{1.7427}$	R^2 = 0.9964
Pi	y = 5.2236ln(x) - 15.787	R^2 = 0.995	$y = 0.0023x^{1.7556}$	**R^2 = 0.9994**
Pi+Zn1	y = 5.2236ln(x) - 15.787	R^2 = 0.995	$y = 0.0025x^{1.7279}$	R^2 = 1
Pi+Zn10	y = 3.347ln(x) - 9.9693	R^2 = 0.9983	$y = 0.0014x^{1.8374}$	R^2 = 0.999
Pi+Zn50	y = 0.6773ln(x) - 1.6904	R^2 = 0.8668	$y = 0.0406x^{0.7841}$	R^2 = 0.9885
Cit	y = 5.1423ln(x) - 15.414	R^2 = 0.9965	$y = 0.0037x^{1.6523}$	R^2 = 1
Cit+Zn1	y = 5.0611ln(x) - 15.141	R^2 = 0.9978	$y = 0.004x^{1.626}$	R^2 = 0.9996
Cit+Zn10	y = 3.3972ln(x) - 10.192	R^2 = 0.9857	$y = 0.0015x^{1.833}$	R^2 = 0.9984
Cit+Zn50	y = 0.565ln(x) - 1.2664	R^2 = 0.9535	$y = 0.0406x^{0.7841}$	R^2 = 0.9885

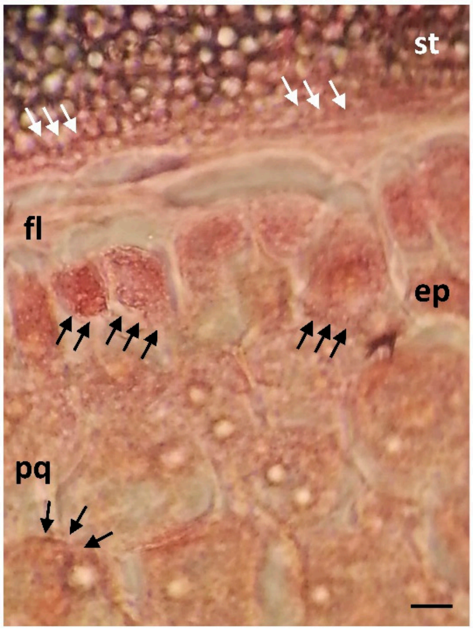

Figure S1. Location of endogenous zinc in the scutellum imbibed in H_2O+H^+. Symbols: ep, epidermal cells; fl, fibrous layer; pq, parenchyma cells; st, starchy endosperm;→, black or white arrows indicate increased staining caused by zinc deposition. Bar: 50 µm.

Methods Supplement 1. Localization of Endogenous Zinc

The endogenous localization of zinc was performed by dithizone staining (DTZ: diphenyl thiocarbazone), which produces a red-purple zinc–dithizonate complex (McNary, 1954; Ozturk *et al*., 2006). Staining was performed in embryos at 24 h of imbibition in H_2O+H^+. Then, embryos were fixed in 70% ethanol for 24 h, dehydrated and infiltrated in paraplast to obtain sections of 8 to 10 µm in thickness, and adhered to glass slides previously covered with 1% gelatine (Ruzin, 1999). The paraplast was extracted from the sections (by extraction with xylene and absolute ethanol), equilibrated in absolute ethanol for 1 h, and transferred to a fresh solution to dissolve 1,5-diphenyl thiocarbazone , 0.5 mg/ml in analytical-grade pure methanol for 30 min. Finally, the excess reagent was washed with absolute ethanol. For the analysis of the sections, they were hydrated and mounted temporarily, and the histological sections were observed with Nomarski differential interference contrast microscopy under an Axioskop Zeiss microscope.

Results Supplement 1

Section of the scutellum at 24 h of germination in H_2O at pH 6.8 showing the location of zinc in the epidermal cells, which are in the process of elongation to transform into epithelium.

Supplementary References

1. Ozturk, L.; Yazici, M.A.; Yucel, C.; Torun, A.; Cekic, C.; Bagci, A.; Ozkan, H.; Braun, H.J.; Sayers, Z.; Cakmak, I. Concentration and localization of zinc during seed development and germination in wheat. *Physiol. Plant.* **2006**, *128*, 144–152.
2. McNary, J.R.; William, F. Zinc-dithizone reaction of pancreatic islets. *J. Histochem. Cytochem.* **1954**, *2*, 185–195.
3. Ruzin, E.S. *Plant Microtechnique and Microscopy*; Oxford University Press: Oxford, 1999.
